# A role for BRCA1 in sporadic breast cancer

**DOI:** 10.1038/sj.bjc.6600863

**Published:** 2003-04-15

**Authors:** J A Fraser, J R Reeves, P D Stanton, D M Black, J J Going, T G Cooke, J M S Bartlett

**Affiliations:** 1 University Department of Surgery, Glasgow Royal Infirmary, 10 Alexandra Parade, Glasgow G31 2ER, UK; 2 Beatson Institute for Cancer Research, Switchback Road, Bearsden, Glasgow, Scotland, UK; 3 Department of Pathology, Glasgow Royal Infirmary, 10 Alexandra Parade, Glasgow G31 2ER, UK

**Keywords:** BRCA1 protein, sporadic breast cancer, immunohistochemistry

## Abstract

To test the hypothesis that altered expression of BRCA1 protein may play an important role in sporadic breast cancer development, 50 randomly selected primary breast cancers (frozen sections, 5 years' median follow-up) were immunolabelled with two monoclonal BRCA1 antibodies (MS110 and MS13). MS110 labelling was exclusively nuclear showing no relation to outcome or tumour pathology. Western blotting demonstrated crossreactivity, suggesting antibody nonspecificity. MS13 labelling was predominantly cytoplasmic. Intense labelling predicted decreased overall survival (*P*=0.012), disease-free survival (*P*=0.029), oestrogen receptor negativity (*P*=0.0004) and c-*erb*B-2 overexpression (*P*=0.006). Western blotting detected a 110 kDa molecule consistent with BRCA1 Δ11b splice variant. BRCA1 protein is postulated to function as a tumour suppressor. We demonstrate cytoplasmic localisation in sporadic breast cancer suggesting excess Δ11b splice variant production, reduced production of full-length BRCA1 and thus postulate reduced tumour suppressor activity. BRCA1 protein appears to have a significant role in both sporadic and hereditary breast cancers.

Approximately 90% of extensive breast and/or ovarian cancer families have loss of function germline mutations in the tumour suppressor genes *BRCA1* or *BRCA2* (17q21 and 13q12–q13, respectively). Germline mutations in *BRCA1* account for about 3% of all breast cancers, with a further 2% resulting from mutations in *BRCA2*. Breast cancer patients with mutated *BRCA1* frequently demonstrate loss of the corresponding wild-type allele (Cornelis *et al*, 1995). Fibroblasts expressing *BRCA1* antisense RNA, with correspondingly low levels of endogenous BRCA1 protein, display accelerated growth and higher tumorigenicity in nude mice than their control counterparts (Rao *et al*, 1996). Retroviral transfection of wild-type *BRCA1* inhibits growth of breast and ovarian cancer cells *in vitro* but not those derived from colon or lung (Holt *et al*, 1996). Since these cell lines are raised from sporadic cancers, these data provide strong support for a role for BRCA1 in sporadic breast cancer. Mutant *BRCA1* transfection does not inhibit breast cancer cell growth; ovarian cancer cell growth is unaffected by 5′ but inhibited by 3′ BRCA1 mutations (Holt *et al*, 1996). In nude mice, MCF-7 breast cancer tumour growth is inhibited by transfection with wild-type but not mutant *BRCA1* (Holt *et al*, 1996). Thus, BRCA1 clearly has an important role in the control of breast neoplasias of sporadic origin.

Wild-type BRCA1 binds to a number of cellular proteins involved in cell cycle regulation including the DNA repair protein Rad51, the tumour suppressor p53, RNA polymerase II holoenzyme, RNA helicase A, CtBP-interacting protein, c-myc, BARD1, BRCA2 protein, etc. BRCA1 is postulated to mediate the involvement of these proteins in DNA repair, transcriptional transactivation and cell cycle control by acting as a converging vehicle for protein association (Scully *et al*, 1997a; Anderson *et al*, 1998; Yu *et al*, 1998; IrmingerFinger *et al*, 1999). By affecting BRCA1 protein tertiary structure, *BRCA1* mutations may thus disrupt complex associations precipitating dysregulation of cellular functions and eventual progression to malignancy.

*BRCA1* germline mutations have been described in only a handful of sporadic breast cancer cases (Futreal *et al*, 1994; Garcia-Patino *et al*, 1998) where they are likely to represent *de novo* mutations. No somatic *BRCA1* gene mutations have been identified without simultaneous germline mutations, unlike ovarian cancer, where single somatic *BRCA1*-truncating mutations have been identified (Merajver *et al*, 1995). BRCA1 protein products may however have an important role in the development of sporadic breast cancers as shown by transfection studies. Increasing evidence exists that abnormal *BRCA1* expression plays a key role in nonhereditary cases, with messenger RNA levels being higher within normal breast epithelium and noncomedo *in situ* breast disease than in cases of sporadic invasive cancer (Thompson *et al*, 1995). Methylation of cytosine residues is a well-recognised explanation for reduced gene expression when no gene mutations or LOH is present (methylation silencing). Indeed, aberrant methylation of the *BRCA1* CpG island promoter at the 5′ end of the *BRCA1* gene has been described and is associated with significantly reduced levels of BRCA1 mRNA (Rice *et al*, 1998), supporting this hypothesis.

With the recent development of BRCA1-specific antibodies (Chen *et al*, 1995; Scully *et al*, 1996), immunohistochemical analysis of tumours is now possible. We have studied 50 randomly selected primary breast cancers by immunohistochemistry using MS110 and MS13 monoclonal antibodies to investigate the relation of BRCA1 expression to pathological, biological and survival parameters.

## METHODS

### Patients

Frozen tumour tissue was obtained from 50 randomly selected primary operable breast cancer cases treated in Glasgow Royal Infirmary from 1984 to 1993. Ages ranged from 40 to 77 years (mean age 60.85) at the time of surgery. Pathology confirmed 47 ductal, two medullary and one lobular carcinoma. Patients were followed up in a dedicated breast clinic (median follow-up 5 years). Follow-up was dated to the last documented clinic visit with all recurrences being confirmed by clinical investigation and, where appropriate, pathology. Conventional pathological data were collected (Table 1

**Table 1:** Relation of MS110 and MS13 immunostaining to pathology (*χ*^2^)

		**[MS110]**		**[MS13]**	
	***N (%)***	***χ^2^***	***P***	***χ^2^***	***P***
*Nodal status*					
Negative	21 (42)	0.246	0.62	0.027	0.87
Positive	28 (56)				
					
*Size (mm)*					
T1 (<21)	16 (33)				
T2 (21–50)	27 (56)	0.404	0.82	0.783	0.68
T3 (>50)	5 (10)				
					
*Grade*					
1	6 (12)				
2	21 (42)	0.679	0.71	4.464	0.11
3	23 (46)				
					
*Oestrogen receptor*					
Negative	22 (48%)	2.14	0.14	12.54	0.0004
Positive	24 (52%)				
					
*NPI*					
1 (<3.4)	7 (14%)	0.860	0.651	0.598	0.742
2 (3.41–5.4)	28 (56%)				
3 (>5.4)	12 (24%)				

). Mortality data were cross-referenced with the regional cancer registry. Normal breast tissue was obtained from seven patients undergoing reduction mammoplasty for use as a negative control.

### Tissue culture

MCF-7, SKOV-3, ZR75-1 and BT20 cells were grown in RPMI and MDA-MB-361, MDA-MB-453, SKBR3 and BT474 cells in DMEM, both supplemented with 10% foetal bovine serum (cell lines available from the American Type Cell Culture Collection). Cells were cultured in a 95% air, 5% CO_2_ humidified atmosphere at 37°C. Cells were grown to near confluence in 175 cm^2^ flasks, harvested by scraping and centrifugation at 100 **g**, and resuspended in 10 ml of PBS (10 mM sodium phosphate, 140 mM sodium chloride pH 7.4). Cells were pelleted at 300 **g** and cell pellets were stored in liquid nitrogen prior to immunohistochemistry.

### Cell fractionation and lysate preparation

Total protein lysates were prepared from 70–100% confluent cells. Cells were recovered by incubation with 0.125% w v^−1^ trypsin, resuspended in RPMI supplemented with 10% FCS, and washed with PBS prior to lysis in modified RIPA buffer (PBS containing 1% Triton X-100, 0.1% SDS and a cocktail of protease inhibitors: 100 *μ*g ml^−1^ phenylmethylsulphonyl fluoride (PMSF), 10 *μ*g ml^−1^ leupeptin, 10 *μ*g ml^−1^ pepstatin, 20 *μ*g ml^−1^ aprotinin and 10 mmol benzamidine). Lysates were passed through a 28-gauge needle, rotated for 20 min at 4°C, and then centrifuged at 15 000 **g** for 20 min to precipitate insoluble cellular debris. Protein yield was estimated spectrophotometrically at 595 nm using the Bradford method (Bradford, 1976).

For cell fractionation, 8–12 175 cm^2^ flasks were collected as above, and washed with PBS. Cells were resuspended in 1.5–2 ml hypotonic buffer (30 mM Hepes, pH 7.5, 5 mM KCl, 1 mM MgCl_2_) and protease inhibitor cocktail as above. After a 30-min incubation on ice, cells were homogenised with a tight-fitting dounce homogeniser and lysis was monitored by microscopy. An equal volume of NP-40 lysis buffer (10 mM Tris, pH 7.4, 250 mM sucrose, 1.0 mM MgCl_2_, 0.1% NP-40) was added to complete lysis. Nuclei were collected by centrifugation at 3000 **g**, washed twice in NP-40 lysis buffer, and then lysed in modified RIPA buffer. Both nuclear and cytoplasmic fractions were centrifuged at 15 000 **g** to precipitate insoluble cellular debris.

### Tumour protein extraction for immunoblotting

Tumours were selected from the highest and lowest MS13 scoring groups. Tumour blocks were maintained at −20°C or lower. Dismembranation was achieved by pulsing for 8 × 1 min at 2000 cycles min^−1^ (Mikro Dismembrator, Braun Biotech International). Chilled modified RIPA buffer (500 *μ*l) was added and the mixture pulsed for a further 2 min. The mixture was then mixed for 30 min at 4°C before being centrifuged at 15 000 **g** to pellet insoluble cellular debris. Protein yield was estimated as previously described. Supernatant was collected and stored frozen as whole-cell lysate.

### Immunoblotting analysis

Immunoblotting was performed on breast carcinoma cell lines MCF-7 and SKOV-3 and tumour lysates. In total, 100 *μ*g of each cellular protein lysate (whole cell, nuclear or cytoplasmic) was denatured by boiling for 10 min in Laemmli buffer and then run on 6% SDS–polyacrylamide gel. Proteins were transferred by electrophoresis at 15 V for 120–180 min to Bio-rad Sequi-Blot PVDF membrane using a Biorad semidry blotter. The membrane was blocked in 5% nonfat milk, 10% sheep serum in Tris buffered saline (TBS: 0.01 M Tris/HCl pH 7.4, 0.15 M NaCl) supplemented with 0.05% Tween 20 (TTBS-milk) overnight at 4°C. The membrane was incubated for 2 h at room temperature with primary antibody in TTBS-milk (antibody concentration of 2 *μ*g ml^−1^ in the case of both MS13 and MS110). Washes were performed in TTBS. Secondary antibody was peroxidase- labelled, affinity purified sheep anti-mouse IgG (Sigma Chemical Company) at a dilution of 1 : 5000 applied for 40 min. The peroxidase signal was developed with ECL as per the manufacturer's directions (Amersham International, Amersham, UK).

### Immunohistochemistry

Frozen section immunohistochemistry was performed as previously reported (Reeves *et al*, 1996). Briefly, 5 *μ*m acetone-fixed cryostat sections of tumour, normal breast tissue or cell line pellets were labelled with monoclonal anti-BRCA1 antibodies MS110 and MS13 (Scully *et al*, 1996) (BRCA1 Ab1 and Ab2, respectively, supplied by Oncogene Research Products, Calbiochem Novabiochem (UK) Ltd, Nottingham, UK). All steps were performed at room temperature in a humidified cabinet. Endogenous biotin was suppressed prior to immunohistochemical labelling, and nonspecific binding was further blocked by preincubating the sections with 25% of both rabbit and human serum in PBS. Sections were incubated with primary antibody for 2 h (antibody concentration of 1 *μ*g ml^−1^ in both cases). Antigen was detected with biotinylated rabbit anti-mouse immunoglobulins (Dako, High Wycombe, UK) applied for 30 min at a dilution of 1 : 400 prior to using the streptavidin–biotin–peroxidase complex detection system (Dako, High Wycombe, UK) according to the manufacturer's instructions. In all, 10% each of human and rabbit serum were included in the primary and secondary antibody diluent. The peroxidase signal was developed with a 10-min exposure to 0.07% nickel chloride, 0.025% diaminobenzidine tetra hydrochloride and 0.01% hydrogen peroxide producing a black precipitate. The nuclei were counterstained with safranin before dehydration and mounting. Negative controls were performed on parallel sections by using isotype-matched nonimmune immunoglobulin in place of the primary antibody.

To confirm the specificity of the MS13 and MS110 antibody, the epitope to which they had been raised was synthesised. This protein fragment competitively inhibited antibody binding in tissues known to give positively labelled results.

### Immunohistochemical scoring

Two different methods of immunohistochemical scoring were utilised because of the differences in staining patterns. MS110 staining was nuclear and so a conventional scoring method was employed, estimating the percentage of labelled tumour cell nuclei. MS13 staining was however cytoplasmic and thus a histoscore was calculated from the sum of (1 × % weakly positive)+(2 × % moderately positive)+(3 × % strongly positive) with a maximum of 300. The mean of the two observers' scores was used for analysis.

### Statistics

BRCA1 scores for each antibody were treated as binary variables (above or below the median score). Tumours with higher than median and lower than median MS110 or MS13 scores were assessed in relation to the following biological variables: nodal status, Bloom and Richardson tumour grade, oestrogen receptor status and tumour size using *χ*^2^ analysis and with the continuous variable age using the Mann–Whitney test. Life table analysis of overall and disease-free survival was performed using Kaplan–Meier estimates and log-rank tests. Spearman's rank correlation was used for direct comparison of the scores with the two antibodies on the tumour samples.

Analysis of agreement for each set of measurements was conducted using the methods of Bland and Altman (1986), (1999). The mean difference between measurements was obtained and 95% confidence intervals for the mean calculated. If these confidence intervals span zero, it is judged that there is no systematic bias measurements in between observers.

## RESULTS

### MS110 immunohistochemistry

**Figure 1:**
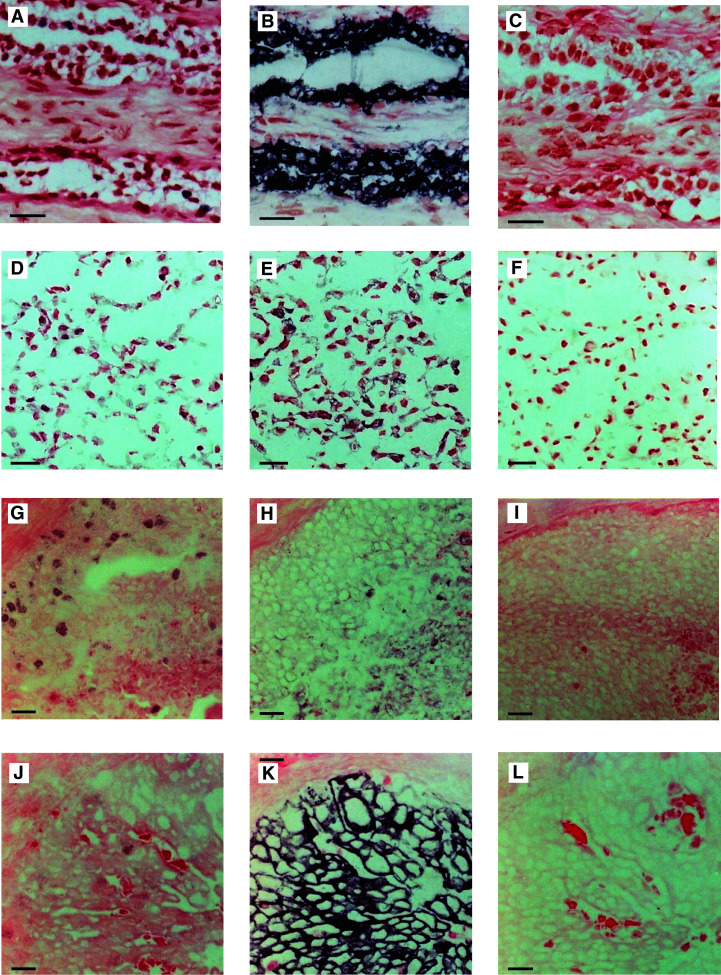
(**A**) Normal breast epithelium labelled with MS110 demonstrating nuclear staining of approximately 1–5% of tumour nuclei; (**B**) parallel section stained with MS13 demonstrating strong cytoplasmic staining of the ductal and terminal ductal lobular unit; (**C**) parallel control section; (**D**) SKBR-3 cell line section labelled with MS110 showing nuclear labelling; (**E**) a parallel section stained with MS13 demonstrating cytoplasmic staining (histoscore 140); (**F**) parallel control section; (**G**) sporadic breast cancer section labelled with MS110 (estimated 30% labelling of the tumour nuclei); (**H**) parallel section stained with MS13 (mean histoscore 80); (**I**) parallel control section; (**J**) sporadic breast cancer section labelled with MS110 (estimated 5% labelling of the tumour nuclei); (**K**) parallel section stained with MS13 (mean histoscore 270); (**L**) parallel control section. Control sections were labelled with isotype-matched nonimmune immunoglobulin in place of the primary antibody. Bars=20 *μ*M.

In histologically normal epithelium from breast reduction specimens MS110 revealed nuclear labelling in approximately 1–5% of epithelial cells (Figure 1A

). The same staining pattern was present in cell lines (nuclear labelling range 2–50%) (Figure 1D and Table 2

**Table 2:** Immunohistochemical data for cell lines

**Cell line/tumour**	**MS110 % +ve nuclei**	**MS13 histoscore**
MDA-MB-361	25	10
MDA-MB-453	2	125
ZR75-1	5	0
MCF7	15	0
SKBR3	50	140
BT20	20	80
BT474	40	25
SKOV-3	40	5

) and in sporadic breast tumour specimens (nuclear labelling ranging ⩽1–60%) (Figures 1G, J). No labelling was present in the parallel control sections (Figures 1C, F, I, L). Of the two independent observers' (JRR and JJG) scores (Figure 2A

**Figure 2:**
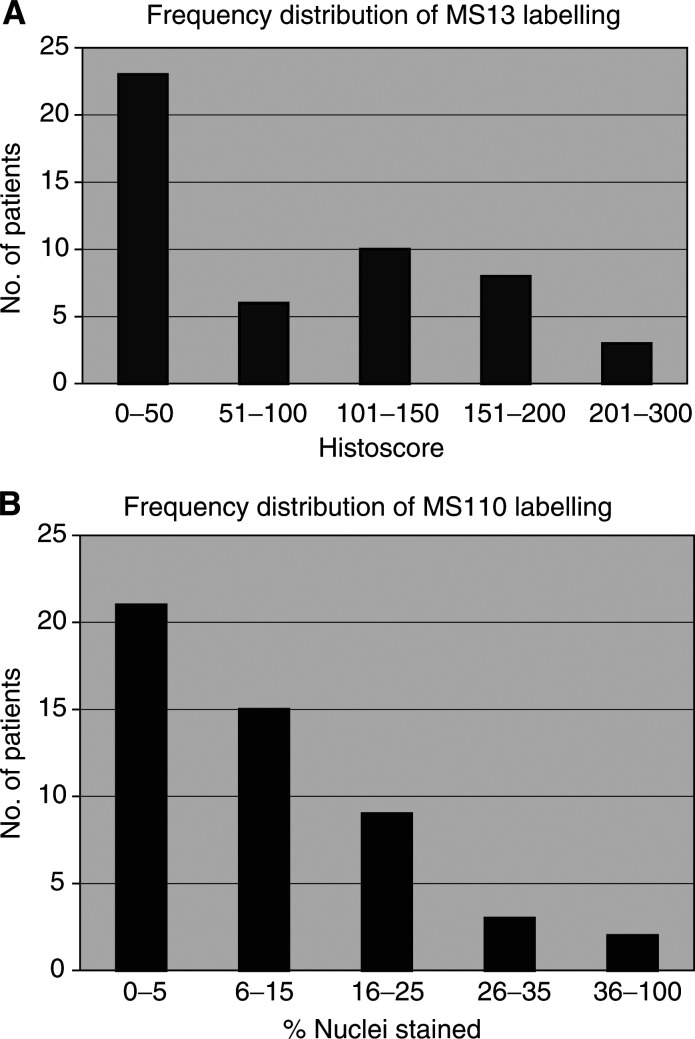
Frequency histograms demonstrating the distribution of (**A**) MS110 labelling and (**B**) MS13 labelling in 50 randomly selected sporadic breast cancers.

), JRR's estimations of the proportion of MS110-labelled nuclei ranged from 1 to 70% with a median of 10%, whereas JJG's scores ranged from 0 to 50% with a median of 5%. For statistical analysis, the mean of both observers' scores was calculated and categorised on a binary basis on either side of the median.

Comparison of MS110 scores between observers produced a mean difference of 5.30. The 95% confidence intervals for the mean do not include zero (2.18–8.42), reflecting a small systematic bias in the scoring (*P*=0.001). Nonetheless, the Pearson correlation coefficient (0.72, *P*=0.0001) and the 95% limits of agreement span a sufficiently small range (−16.68 to 27.28) to enable the two observers' scores to be deemed comparable. Similarly, comparison of MS13 histoscores produced a mean difference of −13.52. The 95% confidence intervals for the mean are −22.65 to −4.39. Once again the confidence intervals do not include zero, reflecting a systematic bias in the histoscoring. The Pearson correlation coefficient is 0.922 (*P*=0.0001) and although the 95% limits of agreement (−77.75 to 50.71) are broad, given the histoscore range of 0–300, they can be considered to demonstrate acceptable interobserver agreement. Again the mean of the two observers' scores was used for analysis.

### MS13 immunohistochemistry

MS13 antibody immunostaining pattern was strong, cytoplasmic and always confined to the ductal and terminal ductal lobular unit epithelial cells in histologically normal breast tissue (Figure 1B). Within all cell lines (Figure 1E and Table 2) and sporadic breast tumour samples (Figures 1H, K), the signal was cytoplasmic with mean histoscores ranging from 0 to 140 and 0 to 257.5, respectively. Parallel control sections of normal and malignant breast samples, and cell lines demonstrated no staining (Figures 1C, F, I, L). Again the mean of the two observers' scores was used for analysis (Figure 2B). JRR's histoscores ranged from 0 to 270 with a median of 50, and JJG's ranged from 0 to 260 with a median of 66. As above, the mean of the two observers' scores was calculated and categorised on a binary basis on either side of the median.

### BRCA1 expression in relation to tumour pathology

Oestrogen receptor positive tumours were significantly more likely to have lower MS13 histoscores (mean histoscore 87.2) than their oestrogen receptor negative counterparts (mean histoscore 136.8) (*P*=0.0004). No significant relation (*P*⩾0.05) was demonstrated between the proportion of MS110 positive nuclei or MS13 histoscore and patient age, nodal status, tumour size, tumour grade or the Nottingham Prognostic Index (Galea *et al*, 1992), which combines the prognostic information given by nodal status, tumour size and tumour grade (Table 1).

A relation was sought between MS110 and MS13 staining patterns and c-*erb*B-2 and epidermal growth factor receptor data previously published by this group (Reeves *et al*, 1996; Robertson *et al*, 1996). A strong relation was evident between intensity of MS13 labelling and increased c-*erb*B-2 expression (*P*=0.006). This was confirmed by fluorescent *in situ* hybridisation (FISH) where high MS13 scorers correlated with the FISH positive expressors (*P*=0.03) (in-house data) (Bartlett *et al*, 2001). No relation was demonstrated between epidermal growth factor receptor (EGFR) and either antibody score. Direct comparison of paired MS13 and MS110 histoscores was performed and demonstrated no relation.

### BRCA1 expression in relation to outcome

**Figure 3:**
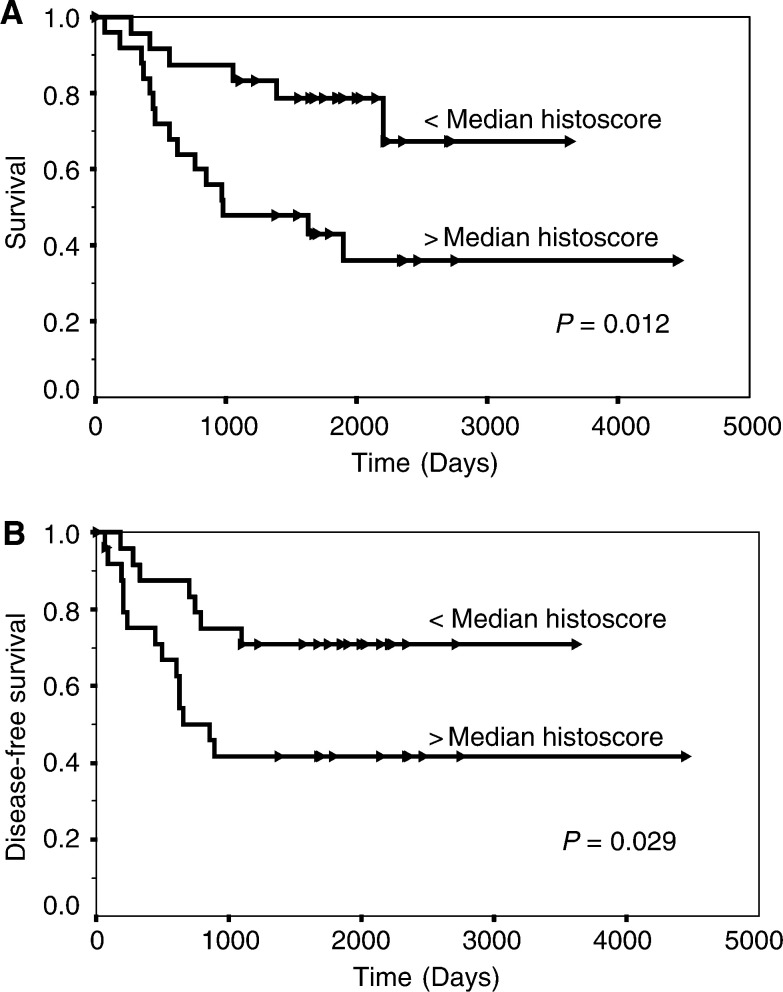
(**A**) Survival curve analysis (from all causes) according to low and high immunohistochemical labelling with the MS13 antibody and (**B**) disease-free survival curve analysis (local and distant recurrence). Arrowheads indicate censored events (Kaplan–Meier estimates and log-rank tests).

Univariate survival analysis was carried out for both sets of data using Kaplan–Meier estimates and log-rank tests. MS110 immunostaining did not relate to disease-free (*P*=0.62) or overall survival (*P*=0.62) in this group. However, high MS13 histoscores were associated significantly with both a shorter overall survival (*P*=0.012; Figure 3A

) and disease-free interval (*P*=0.029; Figure 3B).

### Competitive inhibition of BRCA1 labelling

Differing concentrations of the 304 amino-acid protein fragment were used. Staining remained present at lower concentrations (⩽53 *μ*g ml^−1^), but disappeared as epitope concentrations were increased (absent at ⩾70 *μ*g ml^−1^).

### Western blotting analysis

Western blots were carried out to ensure that both antibodies used for immunohistochemistry were sensitive and specific for BRCA1.

**Figure 4:**
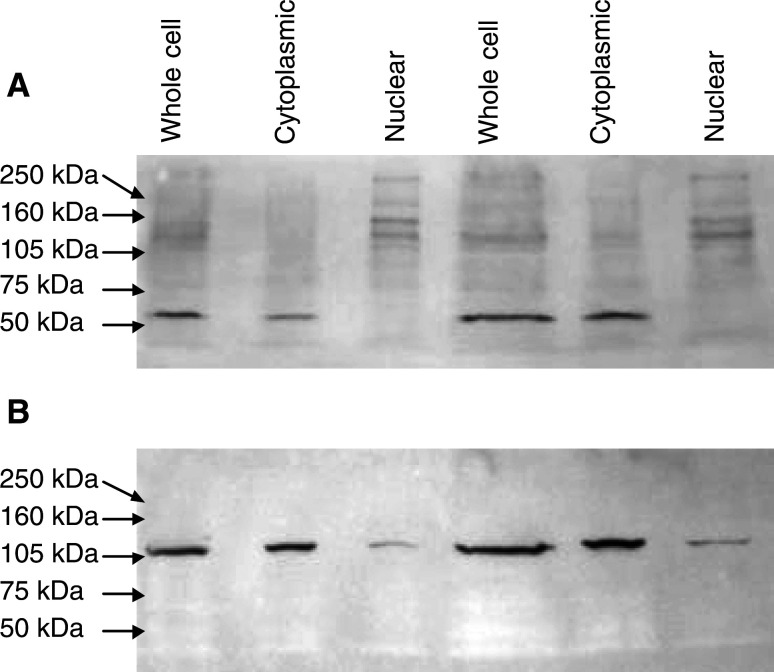
SKOV-3 whole, cytoplasmic and nuclear fractions (100 *μ*g aliquots lanes 1 – 3, and 200 *μ*g aliquots lanes 4–6) probed with (**A**) MS110 demonstrating multiple bands of various molecular weights and (**B**) MS13 demonstrating one band of 110 kDa in the whole cell and cytoplasmic fractions and faintly in the nuclear fraction.

MS110 detected both 220 and 110 kDa bands in the whole cell and the nuclear fractions of both cell lines tested as well as numerous other bands, suggesting that, although detecting the full-length and splice variant of BRCA1, MS110 crossreacts with a number of other proteins (Figure 4

).

MS13 detected a 110 kDa band in the whole cell and cytoplasmic fractions of the cell lines tested, with a faint shadow also in the nuclear fraction, consistent with it detecting the Δ11b splice variant (Figure 4). A faint 220 kDa band could also be detected in whole-cell lysates.

**Figure 5:**
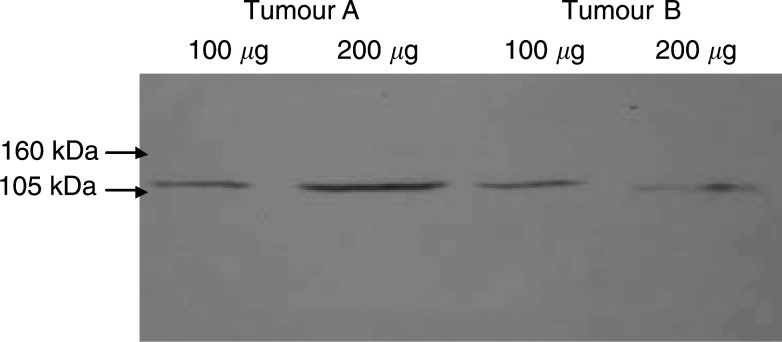
Sporadic breast cancer tumours (tumour A lanes 1 and 2, tumour B lanes 3 and 4). Whole-cell protein lysate (100 and 200 *μ*g aliquots) probed with MS13 demonstrating the presence of one detected molecule of 110 kDa.

Low- and high-scoring tumours from the MS13 immunostained group were selected and protein was extracted for Western blotting. This confirmed MS13 to label a 110 kDa molecule in the tumour setting, consistent with the cell line Western blot findings (Figure 5

). MS110 detected numerous bands ranging from 286 to 54 kDa, an identical picture to that seen with the cell lines.

## DISCUSSION

The *BRCA1* gene and its protein products have been the subject of intensive investigation over recent years because of their proven role in hereditary and putative role in sporadic human breast and ovarian cancer. Controversy concerning the subcellular localisation of the BRCA1 protein has abounded, with it being reported as nuclear (Scully *et al*, 1996), nuclear in normal but cytoplasmic in breast and ovarian carcinoma cells (Chen *et al*, 1995), membrane associated on the cytoplasmic aspect of nuclear invaginations (Coene *et al*, 1997), or a secreted growth inhibitor (Holt *et al*, 1996; Jensen *et al*, 1996). This debate has been fuelled by concerns regarding the specificity of the monoclonal antibodies available for BRCA1 detection (Wilson *et al*, 1996; Thakur *et al*, 1997).

MS110 and MS13 are monoclonal antibodies raised to the N-terminal 304 amino acids of BRCA1 (Scully *et al*, 1996) supposedly specific to BRCA1. Data presented here demonstrate different staining patterns for these two antibodies in frozen breast cancer sections, suggesting that these antibodies recognise distinct protein species or isoforms of the same protein. Staining with both antibodies was successfully competitively inhibited using the 304 amino-acid N-terminus to which the antibodies had been raised, confirming the ability of MS110 and MS13 to detect the BRCA1 N-terminus but not ruling out crossreactivity with confounding proteins.

Western blot analysis, following full optimisation of the conditions, demonstrated MS13 to be specific for a 110 kDa protein consistent with the previously reported Δ11b splice variant of BRCA1 (Wilson *et al*, 1997), with occasionally a 220 kDa molecule faintly demonstrable in cell extracts. No crossreactivity with other protein species was observed. Furthermore, the 304 amino-acid BRCA1 N-terminus was readily identified on Western blot using MS13. In contrast, while MS110 also detected a 110 and a 220 kDa product, numerous other proteins were also detected, suggesting significant crossreactivity with non-BRCA1 protein species. MS110 did, however, also readily identify the 304 amino-acid N-terminus of the BRCA1 protein in Western blot analysis. These data support the conclusion that MS13 is specific for BRCA1 detecting the BRCA1 Δ11b splice variant in a highly specific manner, while MS110 lacks specificity in Western analysis. In the light of these findings, staining with the Ab1 antibody cannot be clearly linked with BRCA1 expression.

Previous studies using anti-BRCA1 antibodies have described crossreactivity with various different molecules (Wilson *et al*, 1996). In this study, EGFR immunohistochemistry was performed in parallel with MS13 and MS110 immunohistochemistry. EGFR staining patterns were entirely distinct from those observed with either MS110 or MS13. Furthermore, although an association was observed between the intensity of MS13 immunostaining and high c-*erb*B-2 expression, the staining pattern for c-*erb*B-2 was also distinct from that of MS13. Additionally, two tumours with previously demonstrated c-*erb*B-2 gene amplification and very high c-*erb*B-2 protein expression (Reeves *et al*, 1996; Robertson *et al*, 1996) were present in the patient cohort and were entirely negative by MS13 immunohistochemistry. These data suggest that MS13 does not crossreact with these members of the type one growth factor receptor family.

The Δ11b splice variant lacks the majority of exon 11 (amino acids 263–1365) and the resultant 110 kDa protein lacks the nuclear localisation signal. This protein is thought to accumulate in the cytoplasm (Thakur *et al*, 1997; Wilson *et al*, 1997). Synthesis of full-length BRCA1 protein is rapidly followed by translocation to the cell nucleus. Despite Western analysis of MS110 demonstrating crossreactivity with a number of protein species, both nuclear and cytoplasmic in localisation, IHC staining with MS110 is almost exclusively nuclear. Although crossreactivity with a non-BRCA1 nuclear located protein in IHC cannot be excluded. It is probable that the MS110 antibody demonstrates stronger affinity for the full-length protein in its native, full-length form.

Cell fractionation studies described here confirmed the 110 kDa protein species detected by MS13 to be predominantly localised in the cytoplasmic cell fractions, thus confirming the immunohistochemical results. The faint presence of the 110 kDa molecule in the nuclear fraction is likely to represent contamination of the nuclear fraction at the time of preparation. Alternatively, a further as yet unrecognised nuclear localisation signal could exist, allowing some translocation of the Δ11b splice variant to the nucleus. Further studies on the subcellular localisation of the Δ11b splice variant would be required to address this.

In this study, immunohistochemistry and Western blotting results for MS13 are concordant. The MS110 antibody is clearly nonspecific, and results should not be extrapolated to BRCA1. MS13 staining is also consistent with the lack of a nuclear translocation signal. Hence, three strands of evidence confirm our results to be correct and reproducible under different fixation conditions. Previous work has suggested that MS13 staining is predominantly nuclear and represents the full-length protein (Wilson *et al*, 1999). However, recent immunoblotting data from the same group (http://www.med.ucla.edu/ora/manuscripts.htm) showed MS13 to detect the Δ11b splice variant in the cytoplasm as well as the full-length BRCA1 species in the nucleus. Our failure to detect nuclear MS13 staining may represent lower concentrations of this protein than levels of splice variant detected in the cytoplasm.

A significant relation was demonstrated between MS13 labelling and overall survival (*P*=0.012) and disease-free survival (*P*=0.029), with increasing intensity of staining indicating worsening outcome in both situations (Figure 3A, B). Intense MS13 staining was also significantly associated with high c-*erb*B-2 expression (*P*=0.006) and oestrogen receptor negativity (*P*=0.0004), both indicators of a poorer prognosis. Familial breast carcinomas with germline mutations of BRCA1 are frequently associated with absence of c-*erb*B-2 expression (Lakhani *et al*, 2002). However, care should be taken in extrapolating findings from cases with mutational alterations to BRCA1 to those with potentially physiologically relevant variations in expression of different isoforms. Our study suggests that sporadic breast cancers with overproduction of the Δ11b splice variant are commonly associated with overexpression of c-*erb*B-2. Oestrogen and progesterone have both previously been reported to induce BRCA1 expression in breast cancer cell lines containing these nuclear hormone receptors (Gudas *et al*, 1995). Initially thought to be a direct response to oestrogen stimulation, increased BRCA1 expression has now been demonstrated to be a response to the increased DNA synthesis initiated by oestrogen stimulation (Marks *et al*, 1997). The function of nuclear BRCA1 as a cell cycle checkpoint regulator has, however, been suggested to be a response to DNA aberrations requiring repair rather than a direct response to DNA synthesis itself (Marquis *et al*, 1995; Scully *et al*, 1997b). It is possible that the type I tyrosine kinase growth factor receptor c-*erb*B-2 induces BRCA1 transcription by a similar pathway. This would suggest that the increase in cell proliferation with associated genetic mutations common to all tumours should cause an increase in BRCA1 transcription and translation. This study would suggest that in sporadic breast cancer cases the overproduced BRCA1 species is the Δ11b splice variant.

No function has as yet been ascribed to the cytoplasmically located Δ11b splice variant; however, the fact that analyses have shown that elimination of exon 11 by differential splicing also occurs in the mouse (Hakem *et al*, 1996) is strong evidence that this message may encode a protein of physiological importance. *In vivo* work has confirmed the Δ11b splice variant to be transcribed at physiologically significant levels (Gudas *et al*, 1995, 1996). Significant data exist suggesting that the Δ11b splice variant is functionally distinct from full-length BRCA1 in several respects. (i) As a result of splice elimination of the NLS encoded in exon 11b, the Δ11b protein cannot autonomously translocate to the nucleus. (ii) The BRCA1 Δ11b splice variant does not exhibit the cell toxicity apparent with overexpression of the full-length protein in transiently transfected cells. (iii) The Δ11b splice variant mRNA has been reported to be differentially reduced or absent in breast and ovarian tumour cell lines relative to exon 11b and transcripts with and without exons 9 and 10 (Wilson *et al*, 1997). All would support a function for the BRCA1 Δ11b splice variant distinct to that of full-length BRCA1.

Altering the intracellular localisation of the BRCA1 species may act as an important physiological regulatory mechanism. In the normal setting, DNA synthesis and genetic mutations are relatively low. A correspondingly low level of full-length BRCA1 compared to splice variant would thus be expected as was found in normal breast epithelium in our data. Similarly, an increase in full-length BRCA1 production could be expected to deal with the increased number of genetic mutations occurring in the malignant setting. In sporadic breast cancer however, it appears that an excess of BRCA1 Δ11b splice variant is produced, perhaps signifying the malfunction of this physiological control mechanism.

No significant correlation was observed with any of the other biological or pathological markers studied (nodal status, tumour size, tumour grade), suggesting that increased levels of Δ11b splice variant are an independent marker of poor prognosis. This is currently under investigation in a larger cohort of patients in our laboratory.

This study demonstrates that MS13 labelling is associated significantly with poor prognosis. MS13 appears to preferentially label the Δ11b splice variant of BRCA1. It would therefore appear that presence of the Δ11b splice variant is a strong negative prognostic marker in sporadic breast cancer. These data demonstrate the importance of the BRCA1 regulatory system in sporadic tumours. No significant association between MS13 histoscore and the Nottingham Prognostic Index (Galea *et al*, 1992) is seen in this series. Although performed in a limited series of cases, we are able to hypothesise from these data that alterations to BRCA1 function, other than inherited mutational changes, may play a significant role in the pathophysiology of breast cancer. We are currently testing this hypothesis in a wider patient cohort to evaluate the potential of MS13 immunohistochemistry as an independent prognostic marker of patient outcome. Should this research confirm our preliminary data, evidence will be provided that BRCA1 is a powerful mediator of sporadic breast cancer aggressiveness.
